# Floral volatiles evoke partially similar responses in both florivores and pollinators and are correlated with non-volatile reward chemicals

**DOI:** 10.1093/aob/mcad064

**Published:** 2023-05-23

**Authors:** Rohit Sasidharan, Robert R Junker, Elisabeth J Eilers, Caroline Müller

**Affiliations:** Department of Chemical Ecology, Bielefeld University, Universitätsstraße 25, 33615 Bielefeld, Germany; Department of Biology, Evolutionary Ecology of Plants, University of Marburg, Karl-von-Frisch-Straße 8, 35043 Marburg, Germany; Department of Environment and Biodiversity, University of Salzburg, Kapitalgasse 4-6, 5020 Salzburg, Austria; Department of Chemical Ecology, Bielefeld University, Universitätsstraße 25, 33615 Bielefeld, Germany; CTL GmbH Bielefeld, Krackser Straße 12, 33659 Bielefeld, Germany; Department of Chemical Ecology, Bielefeld University, Universitätsstraße 25, 33615 Bielefeld, Germany

**Keywords:** Floral volatile organic compounds, insect visitors, neuroethology, electrophysiology, floral rewards, floral toxins

## Abstract

**Background:**

Plants often use floral displays to attract mutualists and prevent antagonist attacks. Chemical displays detectable from a distance include attractive or repellent floral volatile organic compounds (FVOCs). Locally, visitors perceive contact chemicals including nutrients but also deterrent or toxic constituents of pollen and nectar. The FVOC and pollen chemical composition can vary intra- and interspecifically. For certain pollinator and florivore species, responses to these compounds are studied in specific plant systems, yet we lack a synthesis of general patterns comparing these two groups and insights into potential correlations between FVOC and pollen chemodiversity.

**Scope:**

We reviewed how FVOCs and non-volatile floral chemical displays, i.e. pollen nutrients and toxins, vary in composition and affect the detection by and behaviour of insect visitors. Moreover, we used meta-analyses to evaluate the detection of and responses to FVOCs by pollinators vs. florivores within the same plant genera. We also tested whether the chemodiversity of FVOCs, pollen nutrients and toxins is correlated, hence mutually informative.

**Key Results:**

According to available data, florivores could detect more FVOCs than pollinators. Frequently tested FVOCs were often reported as pollinator-attractive and florivore-repellent. Among FVOCs tested on both visitor groups, there was a higher number of attractive than repellent compounds. FVOC and pollen toxin richness were negatively correlated, indicating trade-offs, whereas a marginal positive correlation between the amount of pollen protein and toxin richness was observed.

**Conclusions:**

Plants face critical trade-offs, because floral chemicals mediate similar information to both mutualists and antagonists, particularly through attractive FVOCs, with fewer repellent FVOCs. Furthermore, florivores might detect more FVOCs, whose richness is correlated with the chemical richness of rewards. Chemodiversity of FVOCs is potentially informative of reward traits. To gain a better understanding of the ecological processes shaping floral chemical displays, more research is needed on floral antagonists of diverse plant species and on the role of floral chemodiversity in visitor responses.

## INTRODUCTION

Animal-pollinated plants face a trade-off with respect to their flower traits: they need to attract pollinators for their reproduction but at the same time prevent visits by florivorous organisms. Thus, they use a broad repertoire of various structural and, particularly, chemical traits that can act as advertisements and/or defences (Farré-Armengol *et al.*, 2013; Junker, 2016; Stevenson, 2020). Floral chemicals comprise both floral volatile organic compounds (FVOCs), which allow signal transmission over distances, and stored specialized metabolites present in the flowers, namely in the pollen, nectar or petals, which are perceived only on contact (Knudsen *et al.*, 2006; Schiestl, 2010; Palmer-Young *et al.*, 2019; Rivest and Forrest, 2020). Pollinating flower visitors depend on nutritive pollen and nectar and are attracted to these rewards by both chemical and visual cues (Junker and Parachnowitsch, 2015; Borghi *et al.*, 2017). Besides innate preferences, they may associate display cues with floral resources that serve as rewards (Wright and Schiestl, 2009; Essenberg, 2021). These rewards contain proteins, lipids, carbohydrates and micronutrients (Bonvehi and Jorda, 1997; Vaudo *et al.*, 2020) on which pollinators depend to meet their nutrient requirements.

However, these nutritious sources are also exploited by antagonists, such as florivores. It should be noted that there is sometimes a continuous spectrum from mutualistic pollinators to antagonistic florivores, because some pollinators may overconsume costly resources, whereas florivores may be ‘costly pollinators’. Moreover, in natural environments, pollen limitation may occur (Knight *et al.*, 2005; Aizen and Harder, 2007; Bennett *et al.*, 2020), suggesting competition between plants for pollen vectors, such as insects. Given that pollen is costly to produce and is exploited as a food source by a large number of animals, including many antagonists, plants cannot afford to produce unlimited amounts of palatable pollen. Instead, they select for pollen traits that prevent excessive consumption, e.g. by repelling or not attracting florivores and pollen robbers and by using deterrent or toxic chemicals. However, pollinators must remain attracted to floral displays and should not be deterred substantially or killed (Adler, 2012; Lucas-Barbosa, 2016; Stevenson, 2020). The task of a flower to attract mutualistic flower visitors and, simultaneously, remain concealed from antagonistic flower visitors or repel them with volatile chemical displays has been described as the defence/apparency dilemma (Feeny, 1976; Kessler *et al.*, 2013). Both pollinators and florivores pose a pronounced selection pressure on floral signals in this regard (Whitehead and Peakall, 2009; Theis and Adler, 2012; Schiestl, 2015).

Producing complex floral blends under the influence of selection pressures from both groups of flower visitors is, potentially, one strategy to cope with this dilemma. This includes the use of FVOC bouquets containing specific combinations of both pollinator-attractive and florivore-repellent compounds, which could themselves vary in their emission dynamics (Theis *et al.*, 2007; Kessler *et al.*, 2013). Another strategy of plants is to deploy an ‘optimal defence’ throughout their tissues with specialized metabolites acting as repellents, deterrents or toxins being most concentrated in organs or tissues of high value for plant fitness (McKey 1974; Rhoades and Bergdahl, 1981; McCall and Fordyce, 2010). These metabolites may protect the flowers from feeding damage by florivorous insects (McCall and Irwin, 2006), excess pollen removal by pollinators and infections by pathogens (Junker and Blüthgen, 2010; Palmer-Young *et al.*, 2019; Rivest and Forrest, 2020).

Floral scent and pollen chemicals can be diverse in terms of both biosynthetic origin and chemistry (Knudsen and Gershenzon, 2006). In general, insect-pollinated flowers are highly chemodiverse in their floral scent profiles in comparison to wind-pollinated species (Farré-Armengol *et al.*, 2015). Pollen can also be chemodiverse to some extent (Rivest and Forrest, 2020). Chemodiversity can be measured at different levels, with a focus on the number of compounds (= richness), but also by implementing their evenness, as done, for example, in calculations of the Shannon index or other diversity indices, originally developed for biodiversity of species (Allen *et al.*, 2009; Wetzel and Whitehead, 2020; Petrén *et al.*, 2023). Many studies on floral attractiveness and apparency neglect the role of contact chemicals (Raguso, 2008*b*). Our knowledge on the role of contact chemicals as feeding stimulants or deterrents for the different types of flower visitors is still largely limited to social bees.

In general, odour and taste cues of flowers are closely linked from an evolutionary and biochemical perspective in shared chemical pathways (Borghi *et al.*, 2017). Moreover, volatile cues advertising non-volatile floral rewards may allow for an associative learning of FVOCs to corresponding rewards (Dobson and Bergström, 2000; Wright and Schiestl, 2009; Essenberg, 2021; Hauri *et al.*, 2021), provided that the flower is receptive (Knauer and Schiestl, 2015; Knauer *et al.*, 2021). Flower chemicals can also serve other functions, such as photoprotection or structural optimization of the flower (Essenberg, 2021). The measurement of chemodiversity has roots in information theory, and chemodiversity can thus be related to the information conveyed by a compound mixture, owing to the innate nature of its entropy (Shannon and Weaver, 1949, Allen *et al.*, 2009; Zu *et al.*, 2023). Certain volatile organic compound (VOC) mixtures may also be informative, acting as unique signals (Deisig *et al.*, 2006). Alternatively, plants could distort or modify information contained in (F)VOC bouquets by adding or removing compounds (Kessler and Kalske, 2018). The chemodiversity of FVOCs may be related to the chemodiversity of rewards and may also be informative of nutrient content, but this has, to our knowledge, not been explored. Instead, previous studies have looked at the role of individual FVOCs as ‘honest signals’ (Knauer and Schiestl, 2015; Burdon *et al.*, 2020; Haber *et al.*, 2021).

Here, we take the combined approach of a summary of relevant literature and two meta-analyses to understand the diversity and complexity of flower chemicals and ascertain the roles of various chemical displays in impacting the detectability and behaviour of pollinators and florivores. We review and summarize existing literature and highlight potential trade-offs between conflicting processes, such as pollination and florivory, influenced by chemical displays, in addition to limitations in the availability of studies concerning florivory, which could skew our understanding of the two processes. Within our meta-analyses, we address two main questions. First, do responses to FVOCs differ between pollinators (mutualists) and florivores (antagonists) across various plant species? Second, is floral scent chemodiversity correlated with chemical pollen (floral reward) traits? Together, we summarize the available information on floral chemical displays and their importance for flower visitor behaviour, while taking into account the complexity and contextual nature of these displays.

## VOLATILE DISPLAYS: FLORAL SCENT

### Complexity and functions of FVOCs

FVOCs are a highly diverse group chemically (Knudsen *et al.*, 2006). The complex floral scent generally comprises a mixture of FVOCs emitted at unique ratios and at temporally highly dynamic rates (Schiestl and Ayasse, 2001; Knudsen *et al.*, 2006; Martin *et al.*, 2017; Farré-Armengol *et al.*, 2020). For example, a plant might adapt the timing of FVOC emission to daytimes with the highest pollinator activity and/or lowest florivore activity (Theis *et al.*, 2007; Majetic *et al.*, 2015). Moreover, the scent of flowers often changes soon after pollination (Schiestl and Ayasse, 2001; Bhattacharya and Baldwin, 2012; Proffit *et al.*, 2018). Thus, even during the lifespan of an individual, FVOC emission can be remarkably versatile. Besides the production and emission of FVOCs directly from plant tissues, yeasts and other microbes associated with the flower contribute greatly to the odour blend in the form of their own volatiles or by modifying the plant odour (Helletsgruber *et al.*, 2017; Rering *et al.*, 2018; Schaeffer *et al.*, 2019; Cullen *et al.*, 2021; Francis *et al.*, 2021). Such microorganisms thus add to the chemodiversity of FVOCs (Farré-Armengol *et al.*, 2016).

Compounds produced and/or emitted from flowers are not limited to this organ. Instead, there exists a high degree of convergence in the production of FVOCs and VOCs from vegetative plant tissues (Andersson *et al.*, 2002; Knudsen *et al.*, 2006; Abel *et al.*, 2009; Eilers *et al.*, 2021). However, the diversity of vegetative VOCs is believed to be lower than that of FVOCs (Knudsen *et al.*, 2006). FVOC diversification towards different pollinator species might have led to a greater diversity and scent specialization (Knudsen *et al.*, 2006; Schiestl, 2010). Furthermore, flowers partly produce FVOCs that are identical to compounds involved in animal communication. In fact, an 87 % overlap of FVOCs and VOCs produced by insects has been described (Schiestl, 2010). Given the existing overlap between floral and insect scents, it is hypothesized that the specialization of FVOCs occurred through classes of compounds that were already detectable by insects (Schiestl, 2010). Although pollinators are believed to exert the major selection pressure driving the evolution of FVOCs (Knudsen and Gershenzon, 2006; Raguso, 2008*b*; Schiestl and Johnson, 2013; Schiestl, 2015), evidence is increasing that florivores also impose a conflicting selection pressure on FVOCs (Strauss and Whittall, 2006; Schiestl, 2010, 2015; Junker, 2011). These different selection pressures might have resulted in a complex sets of FVOCs, which are limited by biosynthetic and phylogenetic constraints (Knudsen and Gershenzon, 2006; Schiestl, 2010).

FVOCs play multifaceted ecological roles (Junker, 2016; Farré-Armengol *et al.*, 2020; Cullen *et al.*, 2021; Francis *et al.*, 2021). Apart from attracting (or repelling) pollinators and floral herbivores, certain FVOCs may also attract predators and parasitoids of herbivores. Moreover, some FVOCs can repel herbivores feeding on vegetative parts or induce resistance to antagonists in neighbouring plants (Farré-Armengol *et al.*, 2020; Sasidharan and Venkatesan, 2020). Given that most flower visitors do not specialize on only one plant species, the composition of FVOCs shows great interspecific convergence among plants and is therefore not taxonomically characteristic above the genus level (Knudsen *et al.*, 2006; Schiestl and Johnson, 2013; Farré-Armengol *et al.*, 2020). In fact, some FVOCs are emitted by a huge number of different plant species, of which the monoterpenoids linalool and ocimene and the benzenoid benzaldehyde are among the most commonly reported in both ubiquity and predominance (Knudsen *et al.*, 2006; Farré-Armengol *et al.*, 2020).

### Physiological and behavioural responses to FVOCs by flower-visiting insects

Floral scent must be perceived by insect visitors against the background of other plant volatiles (Raguso, 2008*a*). In most insect species, odour-sensitive sensillae are located on the antennae and on the maxillary palps. These sensillae are hair-like structures that contain olfactory receptor neurons, also called olfactory sensory neurons, and non-neuronal support cells embedded in lymph fluid and sheathed by a porous cuticle (Schmidt and Benton, 2020). The olfactory receptor neurons allow detection and discrimination of individual molecular structures (Bruce and Pickett, 2011). Based on morphological characteristics, such as length, width, cuticle thickness and the number and size of pores, different types of sensillae exist, e.g. basiconic or trichoid sensillae (Schmidt and Benton, 2020). Basiconic sensillae are mostly responsible for detecting odours derived from food such as nectar and pollen, whereas trichoid sensillae allow for pheromone detection (Grabe and Sachse, 2018). Both types are presumed to be responsible for detection of FVOCs in a large number of flower-visiting insects, because FVOCs often resemble scents characteristic of food (such as pollen) or mimic pheromones (Dobson and Bergström, 2000; Raguso, 2008*b*). The electrical signals produced as a result of stimulation of sensillae, i.e. neuronal excitation, are often recorded experimentally with the help of electrophysiological tools, in particular electroantennographic detection, which can be coupled to a gas chromatograph (GC) and mass spectrometer (MS) to reveal the identity of the stimulant compound (Farré-Armengol *et al.*, 2013). Alternatively, other electrophysiological tools, such as single sensilla recordings, can be used (Sokolinskaya *et al.*, 2020).

Behavioural responses of flower visitors to detected FVOCs are governed by the dependence of the insect on floral resources (Junker and Blüthgen, 2010; Farré-Armengol *et al.*, 2013), the order of the insect (Schiestl, 2010; Farré-Armengol *et al.*, 2020) and the chemical class and concentration of the odour (Terry *et al.*, 2007; Junker and Blüthgen, 2010). For example, obligate flower visitors, usually pollinators, were found, on average, to respond positively to FVOCs, whereas facultative, usually antagonistic visitors showed, on average, negative responses. For facultative visitors, monoterpenes, alcohols, ethers and ketones are mainly repellent, whereas benzenoids have often been found to be attractive. Such behavioural observations were mostly based on trap, olfactometer and bait assays, and only some were revealed from toxicity or food choice tests (Junker and Blüthgen, 2010).

Pollinators belonging to different orders generally differ in their preferences for FVOCs from various chemical classes. Such findings were revealed, among others, through modularity analysis of a meta-network of pollinators and associated FVOC classes (Kantsa *et al.*, 2019). For example, coleopteran pollinators generally prefer plants that have more amino acid-derived FVOCs (Farré-Armengol *et al.*, 2020). In a meta-analysis of the overlap between plant and insect VOCs, benzenoids were more commonly associated with lepidopteran pollinators than with hymenopteran ones, although this might not reflect preferences directly (Schiestl, 2010). Nonetheless, benzenoid richness was also found to be higher in plants pollinated by Lepidoptera compared with plants pollinated by species of Coleoptera, Diptera and Hymenoptera (Farré-Armengol *et al.*, 2020). Megachilid bees showed strong positive associations with sesquiterpenes, Apidae with benzenoids and wasps with C6 green-leaf volatiles and some terpenes (Kantsa *et al.*, 2019). Moreover, the effects of FVOCs on flower visitors are concentration-dependent. For example, benzenoids are attractive only at particular ranges of concentration, whereas terpenes may be attractive at lower concentrations but repellent at higher concentrations (Hao *et al.*, 2013).

Previously, few studies have looked into the effects of FVOCs on pollinator and florivore responses within individual plant species, and meta-studies placed importance on comparing insect orders, insect dependence or pollinator groups. To bridge the knowledge gap between pollinators and florivores, below we describe a comparative meta-analysis that we performed on the effects of host plant-derived FVOCs on pollinators and florivores, testing for similarities and differences in their contextual responses.

### Pollinator and florivore responses to FVOCs: first meta-analysis

To explore whether pollinators and florivores differ in their electrophysiological and behavioural responses to FVOCs within the same plant species, we performed a meta-analysis. We began by collecting publications that contained information on FVOCs, searching in the ISI Web of Science (1945–2020), Google Scholar, Google Web Search and the database SCENTbase (for details, see Supplementary data S1) and eventually narrowed down our search to those publications (in total, 55) that reported on electrophysiological and behavioural responses of insects to FVOCs. These included 149 FVOCs tested for responses of 42 pollinator species and 65 FVOCs tested for responses of 40 florivore species. The insect species were classified as pollinators or florivores based on available literature of their activity. For eight plant genera, detection or behavioural responses of both associated pollinators and florivores were tested to the plant-specific FVOCs (Supplementary data Table S1; excluded plant list is given in Supplementary data Table S2). These plant genera were *Brassica* (Brassicaceae), *Cirsium* and *Helianthus* (Asteraceae), *Cucurbita* (Cucurbitaceae), *Daucus* (Apiaceae), *Dichaea* (Orchidaceae), *Fragaria* (Rosaceae) and *Nicotiana* (Solanaceae). For detection and behaviour together, 20 studies were found to report on pollinator responses to FVOCs from these plants, and 19 for florivores, which were combined into the final analysis.

The percentage of electrophysiologically active FVOCs was calculated for pollinators or florivores for each plant genus and responsiveness compared between the two insect groups in total using a χ^2^ test. For the behavioural data, the percentage of FVOCs emitting an attraction, repellence or no response was calculated individually for each plant genus and insect group. Again, attractiveness vs. repellence of FVOCs was compared in total amongst insect groups using a χ^2^ test. Binomial tests were used to examine the distribution of shared attractive and repellent FVOCs between pollinators and florivores. We expected to find a higher number of FVOCs that could be detectable by pollinators compared with florivores, because outcrossing plants can provide diverse and, occasionally, plastic mixtures of compounds (Knudsen and Gershenzon, 2006; Salzmann *et al.*, 2006; Schiestl *et al.*, 2014; Gervasi and Schiestl, 2017). Moreover, pollinators might have specialized on certain floral scents, leading to the ability to detect more FVOCs and to associate them with nutrition in comparison to florivores (Schiestl, 2015). Regarding behavioural responses, we expected to find a higher proportion of FVOCs to be attractive to pollinators when compared with florivores, because plants must minimize trade-offs between pollinator attraction and florivore repellence to maintain the fitness benefits of pollination.

Our meta-analysis revealed the ten most commonly tested FVOCs, which were tested against pollinators and florivores of at least three plant genera for both detection and behavioural responses (Table 1). These are also amongst the most commonly reported FVOCs across angiosperms (Knudsen and Gershenzon, 2006; Farré-Armengol *et al.*, 2020). In total, FVOCs (including unknown compounds) were tested uniquely (against a specific insect) 220 times for pollinators and 102 times for florivores in detection tests from the selected plant genera. Tested pollinators (belonging to 16 species) responded to 151 (69 %) of these tests, while tested florivores (21 species) responded to 83 (81 %) of these tests, which corresponds to 62 and 32 FVOCs, respectively (Table 2). Thus, florivores were significantly more likely to detect tested FVOCs than pollinators (χ^2^ = 5.069, *P* = 0.024). This pattern was found in most plant genera, except for *Cucurbita* and *Fragaria*, which might indicate different specificity across plant taxa. However, studies with both pollinator and florivore detection responses are still limited; thus, the interpretation of these data must be handled carefully.

**Table 1.** Most abundant FVOCs, tested for both detection and behaviour in at least three plant genera, on at least one pollinator and one florivore species. Values represent percentages of the total number (in parentheses) of insect species tested for detection or behaviour. For a given compound, an insect was sometimes tested repeatedly (e.g. *Apis mellifera*), but the response was found to be uniform, hence averaged below.

Compound	Detection		Behaviour					
			Attracted (+)		Repelled (−)		No response (0)	
	Poll	Flor	Poll	Flor	Poll	Flor	Poll	Flor
2-Phenylethanol	77.8 (9)	100 (3)	66.7(3)	33.3 (3)	0 (3)	33.3 (3)	33.3 (3)	33.3 (3)
3-Hexenol	100 (4)	100 (1)	14.3 (7)	14.3 (7)	14.3 (7)	0 (7)	71.4 (7)	85.7 (7)
Benzaldehyde	100 (9)	100 (4)	33.3 (9)	27.3 (11)	0 (9)	0 (11)	66.7 (9)	72.7 (11)
Benzyl alcohol	87.5 (8)	100 (2)	28.6 (7)	20.0 (10)	0 (7)	0 (10)	71.4 (7)	80.0 (10)
Hexanol	100 (3)	100 (2)	0 (2)	50.0 (2)	100 (2)	0 (2)	0 (2)	50.0 (2)
Limonene	66.7 (3)	100 (3)	50.0 (2)	50.0 (2)	0 (2)	0 (2)	50.0 (2)	50.0 (2)
Linalool	90.0 (10)	100 (2)	22.2 (9)	0 (8)	0 (9)	12.5 (8)	77.8 (9)	87.5 (8)
Methyl salicylate	100 (10)	100 (4)	37.5 (8)	0 (9)	0 (9)	11.1 (9)	62.5 (8)	88.9 (9)
Nonanal	100 (3)	100 (1)	50.0 (2)	0 (1)	0 (2)	0 (1)	50.0 (2)	100 (1)
Phenylacetaldehyde	85.7 (7)	100 (6)	28.6 (7)	41.7 (12)	0 (7)	0 (12)	71.43 (7)	58.3 (12)

Abbreviations: Poll, pollinator; Flor, florivore.

**Table 2.** Differences between responses of pollinators and florivores to tested FVOCs of different plant genera. Percentage of FVOC tests that were detected electrophysiologically or that acted as an attractant or repellent in behavioural studies (percentage of *n*) and total number (*n*) of unique FVOC tests with insect species within that category are given. The total represents every unique combination of tested FVOC and insect. Proportions differing significantly (*P* ≤ 0.05, χ^2^ test) for the total between pollinators and florivores are highlighted in bold. No response was included as a category in tests for attractive/repellent FVOCs, but the data are not shown below. Data are from 32 studies in total (for details, see Supplementary data Table S1).

Plant	Response	Pollinator		Florivore		Significance
		Percentage of total	Total *n*	Percentage of total	Total *n*	
*Brassica* spp. (*B. napa* and *B. rapus*)	Detected	91.3	23	100	8	
	Attractive	52.0	25	75.0	4	
	Repellent	4.0		0		
*Cirsium* spp. (*C. arvense* and *C. japonicum*)	Detected	70.4	81	90.9	11	
	Attractive	24.1	54	9.1	66	
	Repellent	0		3.0		
*Cucurbita* spp. (*C. maxima* and *C. pepo*)	Detected	100	2	96.6	29	
	Attractive	66.7	3	30.8	65	
	Repellent	0		1.5		
*Daucus carota*	Detected	23.1	13	75.0	8	
	Attractive	100	3	0	0	
	Repellent	0		0		
*Dichaea pendula*	Detected	0	0	100	1	
	Attractive	0	1	100	1	
	Repellent	0		0		
*Fragaria* spp. (*F. vesca* and *F. × ananassa*)	Detected	100	13	58.3	36	
	Attractive	0	4	50.0	2	
	Repellent	50.0		0		
*Helianthus annuus*	Detected	68.5	54	100	1	
	Attractive	100	2	100	1	
	Repellent	0		0		
*Nicotiana attenuata*	Detected	68.0	50	100	10	
	Attractive	26.1	23	19.1	21	
	Repellent	17.4		28.6		
Total	Detected	**68.6**	220	**81.4**	102	*P* = 0.024 (χ^2^ = 5.069)
	Attractive	33.0	112	22.0	159	*P* = 0.07 (χ^2^ = 5.33)
	Repellent	8.0		5.7		

The fact that florivores seem to physiologically detect a higher percentage of tested FVOCs than pollinators could be an outcome of stronger pollinator specialization on floral scent (Salzmann *et al.*, 2006; Farré-Armengol *et al.*, 2020). Pollinators might thus rely on detecting a more limited set of corresponding FVOCs that evolved under directional selection towards attracting them rather than florivores (Schiestl, 2015). Presumably, florivores might have adapted to the overall FVOC chemodiversity that evolved under selection pressures from both pollinators and florivores, among others (Whitehead and Peakall, 2009; Schiestl, 2010, 2015; Junker, 2011). Moreover, pollinators are often far more mobile than herbivores, including florivores (Theis and Adler, 2012; Underwood *et al.*, 2020). Thus, pollinators can scout over larger distances to find floral resources and, potentially, afford to detect a more limited number of FVOCs. In contrast, florivores cannot afford a prolonged foraging time owing to their limited mobility and might thus need to be responsive to a larger number of FVOCs. Lastly, it is important for florivores to detect sensitively the toxic compounds produced by plants against them, which might also have sensitized them to related FVOCs (Twaij and Hasan, 2022). In contrast, some pollinators might be resistant or tolerant to these compounds (Wright *et al.*, 2010; Barlow *et al.*, 2017).

With regard to behavioural responses, no general differences were observed in the attractiveness of FVOCs to pollinators in comparison to florivores in the tested plant genera. We found 112 unique tests (as an unrepeated combination of a given FVOC tested against a given insect species) of attractiveness to pollinators and 159 tests to florivores. Most of the tests were performed using choice, olfactometer or proboscis extension reflex assays, usually comparing preferences for blank control vs. an FVOC odour (Table 1). Towards several of the ten most commonly tested FVOCs, relatively more pollinator species seemed to be attracted, whereas more florivore species were repelled, although this could not be tested statistically because the numbers of tests were low (Table 1). Among these FVOCs, the terpene linalool is reported to act as a repellent or deterrent to herbivores and florivores in several studies (Theis *et al.*, 2007; Kamatou *et al.*, 2008; Junker *et al.*, 2010*a*). Interestingly, our analysis revealed that an unrelated but commonly occurring benzenoid, methyl salicylate, also seems to play a similar role in attracting floral pollinators while repelling florivores. Although trade-offs between attracting pollinators and repelling florivores are hypothesized to exist (Farré-Armengol *et al.*, 2013), compounds such as linalool and methyl salicylate might offset such trade-offs by causing distinct responses in both groups of flower visitors. One proposed explanation might be that mutualistic pollinators are often obligate flower visitors, whereas antagonistic flower visitors are often facultative, leading to a co-evolutionary pathway where an FVOC is attractive to pollinators in general or repellent to florivores in general (Junker and Blüthgen, 2010). However, these compounds might be either tolerated or toxic to different insect species, depending on their host plant specialization. Alternatively, such distinct responses to individual floral VOCs might be evident when pollinators and florivores belong to different insect orders (Harrewijn *et al.*, 1994; Schiestl, 2010; Farré-Armengol *et al.*, 2020). In our first meta-analysis, the majority of florivores tested in the various studies belonged to the Coleoptera, and a few studies were performed on florivores belonging to Hymenoptera, Lepidoptera, Orthoptera and Hemiptera, whereas the majority of tested pollinators belonged to the Hymenoptera and Lepidoptera and some to the Diptera (Supplementary data Table S1). The phylogenetic status and the functional role in relationship to floral resources (i.e. pollination vs. florivory) might thus be related (Schiestl, 2010). Taken together, it seems that only a few compounds in floral bouquets mediate contrasting responses in some pollinators and florivores. Observations of insect responses are biased towards FVOCs that are more selectively detected by tools such as GC-MS or GC-flame ionization detection. However, more tests are needed on ‘less abundant’ FVOCs. Bioassays with manipulated FVOC bouquets using floral extracts might help to elucidate the role of less abundant FVOCs (Larue *et al.*, 2016).

Although few FVOCs cause contrasting responses, significantly more of the tested FVOCs emitted by the eight plant genera act as attractants rather than repellents to both groups of flower visitors, pollinators and florivores (Table 3). Attractant FVOCs are likely to have evolved primarily to attract pollinators, whereas repellent FVOCs might have evolved under both pollinator- and florivore-imposed selection pressures. Given that florivores eavesdrop on floral cues meant to attract pollinators, attractive (rather than repellent) FVOCs could represent a major source of the trade-off faced by flowering plants. However, the low number of studies, particularly for florivores, makes it difficult to determine a clear separation of pollinator-attractive and pollinator/florivore-repellent FVOCs.

**Table 3.** Total shared, attractive and repellent FVOCs of eight plant genera tested for behavioural responses of pollinators and florivores. Values represent percentages of the numbers (in parentheses) of FVOCs tested. Proportions highlighted in bold indicate significant differences (*P* = 0.04, binomial test) in attractive vs. repellent shared FVOCs.

Plant	Total shared FVOCs tested against both pollinators and florivores	Shared attractive	Shared repellent
*Brassica* spp.	13.6 (22)	66.7 (3)	0 (3)
*Cirsium* spp.	64.3 (14)	33.3 (9)	0 (9)
*Cucurbita* spp.	9.7 (31)	33.3 (3)	0 (3)
*Daucus carota*	0 (3)	0 (0)	0 (0)
*Dichaea pendula*	100 (1)	0 (1)	0 (1)
*Fragaria* spp.	0 (6)	0 (0)	0 (0)
*Helianthus annuus*	0 (5)	0 (0)	0 (0)
*Nicotiana attenuata*	80.0 (20)	6.3 (16)	6.3 (16)
**Total counts**	31.4 (102)	**25.0** (32)	**3.1** (32)

All plant species included in our analysis are outcrossers or at least benefit greatly from outcrossing, and their flowers are visited by a number of different insect taxa. This pattern might well be representative for various plant–insect relationships, because most outcrossing plants are visited by mutualistic and antagonistic insects of different taxa and degrees of specialization. In our meta-analyses, the numbers of detection tests were biased towards pollinators, whereas the numbers of behavioural tests were biased towards florivores. This might have influenced the results, particularly on detection, because more tests might reveal lower overall proportions of FVOC detection. Clearly, there is a need for more studies looking at florivores and their responses to FVOCs in other plant genera, because at present we could gather florivore response data from only eight genera. Responses to FVOCs should also be compared in plants that are pollinated by specialists, such as nursery pollination systems (Dötterl *et al.*, 2006) or in plants that make use of mixed mating and are highly flexible in pollinator attraction.

### Context-dependence of insect responses to FVOCs

Individual flower visitors can respond differently to FVOCs depending on the context. The composition of available odour receptors, hence the number of FVOCs that are detected by receptors, is species-specific. Together with the specificity in neuronal or higher cognitive processes, this can lead to a strong receiver bias (Schiestl and Johnson, 2013). Yet even within the same insect species, the ability to detect a compound and respond behaviourally to it depends on internal and external conditions, such as food supply and stress. In humans, a high degree of chemosensory plasticity exists, depending, for instance, on age, disease state and environmental exposure (May and Dus, 2021). Similar levels of plasticity exist in insects. For instance, starvation induces enhanced olfactory detection and transcriptional changes encoding chemosensory proteins and odorant-degrading enzymes in *Spodoptera littoralis* caterpillars (Poivet *et al.*, 2021). In small-scale diverse natural environments and for flower visitors that differ in age, sex, genetic origin and other factors, odour perception is highly subjective and temporally dynamic, even within the same insect species, forming individual ‘insect odourscapes’ in the vicinity of flowers (Conchou *et al.*, 2019). The difficulty of breaking down this complex picture into simple rules is the main limitation to our understanding of the relationship between FVOCs and the different flower visitors. Beyond this natural plasticity in flower chemistry and visitor behaviour, the presence of anthropogenic pollutants, such as agrochemicals, complicates the relationships, because these can impede the foraging activity of insects (Kessler *et al.*, 2015).

Moreover, single compounds in the mix of emitted FVOCs interact in the odour plume above flowers, and insect responses to a whole blend often differ from responses to individual components (Bruce and Pickett, 2011). Single FVOCs might not be recognized as host cues when perceived outside the context of the entire bouquet. However, FVOCs are often tested individually for electrophysiological detectability and for behavioural responses. In order to elucidate which compounds mask or eliminate the attractive effect of other FVOCs, corresponding mixtures need to be investigated. Further studies are required to understand flower–insect interactions in complex environmental settings in order to account for contextual dependence of responses and plasticity of both FVOCs and insect behaviour. Interaction network analyses of the complex networks between plants, their FVOCs and flower visitors might also help in interpretation of such contextual data, including associations between FVOCs and insect visitor groups, besides the role of abiotic factors in insect responses to FVOCs (Junker *et al.*, 2010*b*; Larue *et al.*, 2016; Kantsa *et al.*, 2019).

## CONTACT CHEMICAL DISPLAYS: NUTRIENTS AND TOXINS IN POLLEN

### Complexity and functions of pollen chemicals

Plant pollen contains 2.5–61 % proteins and 2–20 % lipids (Roulston *et al.*, 2000; Annoscia *et al.*, 2017), and the remaining portion consists mainly of carbohydrates, such as starch (Roulston *et al.*, 2000) and the sugars fructose, glucose and sucrose (Bonvehi and Jorda, 1997). However, the chemical composition of pollen varies greatly depending on plant phylogeny (Roulston and Buchmann, 2000; Vaudo *et al.*, 2020), seasonal appearance and pollination type. For instance, pollen from spring- and autumn-blooming plants contains higher protein proportions compared with pollen from summer-blooming plants (Roulston *et al.*, 2000; Liolios *et al.*, 2015). The pollen protein content is notably higher in pollinator-dependent plants than in plants with low insect dependence (Ruedenauer *et al.*, 2019). Thus, the protein content of pollen is generally considered to be the most important characteristic of quality for pollen-feeding insects. However, a correlation between pollen protein content and insect behavioural preferences is not apparent across different species of plants and flower visitors (Roulston *et al.*, 2000). At least for certain bee species, including the bumblebee *Bombus impatiens*, the honeybee *Apis mellifera* and the hornfaced bee, *Osmia cornifrons*, a clear pattern seems to emerge when focusing on the ratio of the main pollen macronutrient components; they generally prefer pollen with defined protein-to-lipid ratios (Vaudo *et al.*, 2016, 2020). Preferences for certain protein-to-lipid ratios might also exist in other pollen-feeding insects, but more studies are needed to confirm this.

Nutrients in pollen do not act solely as rewards for the purpose of positive reinforcement of pollinators, but also serve an important physiological function in the process of fertilisation of flowers. Pollen proteins enable pollen tube formation (Roulston *et al.*, 2000), and pollen lipids are required to penetrate the stigma (Wolters-Arts *et al.*, 1998). Thus, pollen nutrients must be considered for their multiple functions in both interactions of plants with flower visitors and endogenous purposes.

In addition to compounds of nutritional value, chemicals in pollen and other flower parts also include deterrent or toxic compounds, which belong to the same chemical classes as those produced for defence against herbivores elsewhere in the plant (Rivest and Forrest, 2020; Stevenson, 2020). The most common classes of specialized metabolites in pollen are alkaloids, terpenoids and phenolic compounds, such as flavonoids and phenylpropanoids (Palmer-Young *et al.*, 2019). Some of these may be toxic when present in sufficiently high concentrations (Palmer-Young *et al.*, 2019; Rivest and Forrest, 2020). Toxins in floral tissue deter florivores and can thus reduce floral damage (Adler *et al.*, 2001; Strauss *et al.*, 2004; Irwin and Adler, 2006; Austel *et al.*, 2021). In many of these cases, such compounds simultaneously deter beneficial pollinators (Irwin and Adler, 2006; de Mesquita *et al.*, 2010; Irwin *et al.*, 2014). However, those toxins are also believed to be important in driving the specialization of certain bees as pollinators for plants (Rivest and Forrest, 2020).

### Physiological and behavioural responses to pollen chemicals by flower-visiting insects

For physiological perception of contact chemicals, insect species possess taste organs, so-called gustatory sensillae, mostly on their tarsi, antennae and mouthparts (Mitchell *et al.*, 1999; Bestea *et al.*, 2021). These sensillae are similar in structure to olfactory sensillae; they contain gustatory receptor neurons and non-neuronal support cells embedded in lymph fluid, but the sheathing cuticle is uniporous (Mitchell *et al.*, 1999; de Brito Sanchez *et al.*, 2007; Bestea *et al.*, 2021). By repeated experiences, flower visitors can learn, in a species-specific manner, to associate visual and volatile cues with encountered contact chemicals (Reinhard *et al.*, 2004), such as rewards, deterrents and toxins. However, particularly little is known about how and to what extent non-volatile floral compounds, such as pollen nutrients, can be detected and act as feeding stimulants or deterrents to different flower visitor species (Muth *et al.*, 2016). Taste perception is particularly well studied in honeybees. They were found to respond to sugars, salts, amino acids (Jung *et al.*, 2015) and fatty acids (Ruedenauer *et al.*, 2021), although their antennal gustatory sensillae are less likely to respond to bitter substances (de Brito Sanchez *et al.*, 2005, 2007). In fact, several pollinators might lack gustatory receptors that perceive a bitter taste, but still show an avoidance response to such tastes (Ayestaran *et al.*, 2010; Guiraud *et al.*, 2018), which might be attributable to the inhibition of sweet-responding gustatory receptors (Bestea *et al.*, 2021).

With regard to behavioural responses, preferences of flower visitors are generally investigated for various pollen components. Preferences for protein contents in pollen differ seasonally and depend on the insect species (Liolios *et al.*, 2015; DeGrandi-Hoffman *et al.*, 2021). For instance, honeybees prefer protein-rich pollen late in the season (Quinlan *et al.*, 2021). Pollen amino acid composition may affect the foraging behaviour of honeybees (Cook *et al.*, 2003) but seemingly not of common bumblebee species (Kriesell *et al.*, 2017). Instead, bumblebees use pollen lipid content as a cue to assess overall diet quality and maximize their fitness (Ruedenauer *et al.*, 2020). Furthermore, pollen lipid constituents, such as the free fatty acids oleic acid, α-linolenic acid or linoleic acid, affect foraging behaviour by enhancing the visual learning ability in honeybees and bumblebees (Zarchin *et al.*, 2017; Muth *et al.*, 2018). Moreover, pollen lipids can improve the resilience of bees to pathogen and parasite infestations (Schmehl *et al.*, 2014; Annoscia *et al.*, 2017; Tosi *et al.*, 2017; Dolezal and Toth, 2018; Barraud *et al.*, 2020; Crone and Grozinger, 2021). On the contrary, augmented lipid ingestion increases bumblebee mortality (Ruedenauer *et al.*, 2020). In addition to pollen macronutrients, pollen micronutrients can play a role for flower visitor preferences. For instance, sodium and potassium in pollen influence the preferences of honeybees (Khan *et al.*, 2021).

Fewer studies exist on the effects of nutritional components on florivores. Amino acids such as l-alanine and l-serine were found to evoke strong electrophysiological and behavioural responses in the western corn rootworm (Hollister and Mullin, 1998). Sugars evoke an additive positive behavioural response in this florivorous species (Kim and Mullin, 2007). However, the feeding behaviour and responses to these available nutrients can be altered by the presence of toxic specialized metabolites, as suggested for the facultative florivore *Coleomegilla maculata* (Lundgren and Wiedenmann, 2004).

Toxins in floral tissues can also be of value to flower visitors for defence or communication purposes. For example, pollen toxins can repel or deter parasites, and bees protect their pollen stores with these toxins (Spear *et al.*, 2016). A contribution of the pollen diet for bumblebees by ‘low quality’ (Nicolson and Human, 2013) and flavonoid-rich (Fatrcová-Šramková *et al.*, 2016) sunflower pollen reduces the prevalence of infection with a protozoan pathogen (Giacomini *et al.*, 2021). Likewise, the consumption of nectar alkaloids can reduce the protozoan pathogen load in bumblebees (Manson *et al.*, 2010). Although toxins in pollen are believed to be targeted largely against florivores (Adler *et al.*, 2001; Strauss *et al.*, 2004; Irwin and Adler, 2006; Austel *et al.*, 2021), pollinators may, nonetheless, also be affected by pollen and nectar toxins at certain concentrations, leading to deterrence, impaired development or even death (Irwin and Adler, 2006; de Mesquita *et al.*, 2010; Irwin *et al.*, 2014; Stevenson, 2020). Thus, a similar trade-off as found for FVOCs might exist in pollen traits, namely, the need to protect nutrients serving as rewards for pollinators vs. toxins to deter florivores and excessive pollen consumers.

Potentially deleterious effects of suboptimal pollen nutrient composition might be overcome by mixing low- and high-quality resources (Bukovinszky *et al.*, 2017). Furthermore, most flower visitors mix pollen with nectar, leading to nutritional complementarity (Blüthgen and Klein, 2011). Nectar is an important source of energy and should thus be considered in this context (Nicolson, 2011). Moreover, individual harmful compounds, such as toxins, can be diluted by mixing pollen sources (Eckhardt *et al.*, 2014). However, this strategy of dietary mixing can be used by both groups of flower visitors, pollinators and florivores; thus, such a trade-off might be difficult to resolve for plants.

### Relationships between FVOCs and pollen chemicals: second meta-analysis

FVOCs and pollen chemicals are mostly studied independently, and little is known about potential relationships between FVOCs and other floral chemical traits. Some studies suggest that floral signals are often informative of reward trait quality or composition (Dobson and Bergström, 2000; Essenberg, 2021; Hauri *et al.*, 2021; Zu *et al.*, 2023). Moreover, FVOC production and that of stored toxins could be linked biosynthetically (Pichersky *et al.*, 2006; Dudareva *et al.*, 2013; Twaij and Hasan, 2022). Thus, we expected that the chemodiversity of FVOCs and pollen might be correlated. Flower visitors often respond to blends of FVOCs (Bruce and Pickett, 2011; Kárpáti *et al.*, 2013), and if FVOC chemodiversity is related to pollen chemodiversity, FVOC diversity could inform pollinators or florivores about the nutrient status or toxicity of pollen resources, allowing them to determine the quality at a distance.

We performed a second meta-analysis, using the same literature survey as mentioned above, to test whether FVOC chemodiversity is related to pollen traits, namely nutrient content and chemodiversity of toxins known from pollen. Studies were selected based on the joint availability of data for plant species on the FVOC profile (as a measure of FVOC chemodiversity), their pollen protein content (as a measure of resource quality) and toxins found in pollen (Supplementary data Table S3). As a measure for chemodiversity of FVOCs, we used the compound richness (i.e. number of FVOCs) and the Shannon diversity (*H*_s_ = −Σ *p*_*i*_ × ln *p*_*i*_, where *p* is the relative abundance of an FVOC, *i*) calculated based on the FVOC data from various sources (Supplementary data Table S4) and averaged for the different values for the individual plant species (Supplementary data Table S5). Pollen toxins were grouped into their respective chemical classes (alkaloids, terpenoids etc.). Correlations between the different traits were tested using Pearson’s or Spearman’s rank correlation analyses, depending on whether the data were parametric or not (Supplementary data S1).

**Fig. 1.**
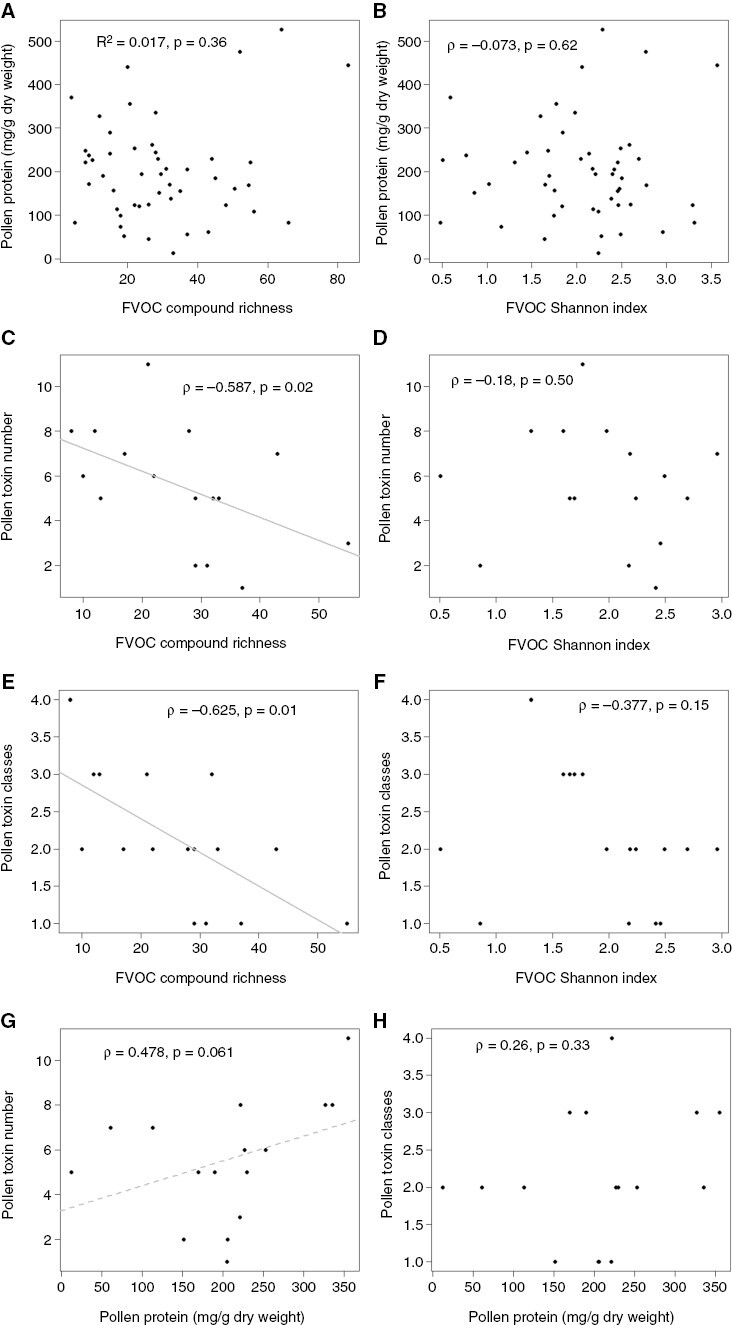
Correlations between FVOC chemodiversity, amount of pollen protein and number of described pollen toxins. Correlation between: (A) amount of pollen protein (in milligrams of protein per gram of pollen d.w.) and compound richness (*n* = 49); (B) amount of pollen protein (in milligrams of protein per gram of pollen d.w.) and FVOC Shannon index (*n* = 49); (C) number of pollen toxins and FVOC compound richness (*n* = 16); (D) number of pollen toxins and FVOC Shannon index (*n* = 16); (E) number of pollen toxin classes and compound richness (*n* = 16); (F) number of pollen toxin classes and FVOC Shannon index (*n* = 16); (G) number of pollen toxins and amount of pollen protein (in milligrams of protein per gram of pollen d.w.) (*n* = 16); and (H) number of pollen toxin classes and amount of pollen protein (in milligrams of protein per gram of pollen d.w.) (*n* = 16). Data were analysed by correlation tests, with *R* (Pearson’s correlation coefficient), ρ (Spearman’s rank correlation coefficient) or *P*-values given (for data, see Supplementary data Table S3).

For 49 plant species, data on FVOC composition and amounts of pollen protein were available (Supplementary data Table S3). Neither the FVOC richness (Pearson correlation, *r*^2^ = 0.017, *P* = 0.36) nor the FVOC Shannon diversity index (Spearman correlation, ρ = −0.073, *P* = 0.62) was correlated with the amount of protein in pollen [weight of protein per dry weight (d.w.) of pollen (in milligrams per gram)] across these studies (*n* = 49; Fig. 1A, B). Sixteen studies were found to report on FVOCs and pollen toxins. Across these studies, compound richness of FVOCs was negatively correlated with the number of stored toxins in pollen within the same plant species (Spearman correlation, ρ = −0.587, *P* = 0.02). However, the Shannon indices of the FVOCs were not correlated with the number of stored toxins (Spearman correlation, ρ = −0.18, *P* = 0.50; Fig. 1C, D). Likewise, the compound richness of FVOCs was found to be negatively correlated with the number of pollen toxin classes (Spearman correlation, ρ* *= −0.625, *P* = 0.01), whereas the Shannon index was not (Spearman correlation, ρ = −0.377, *P* = 0.15; Fig. 1E, F). The negative correlation between the richness of FVOCs and pollen toxins might be informative, such that more chemodiverse flowers are less toxic and therefore attractive to a wider array of flower visitor species. Alternatively, the negative correlation might be a consequence of metabolic or biosynthetic trade-offs between floral volatiles and specialized metabolites stored in pollen. However, such trade-offs might be minimized if compounds are biosynthetically related or chemically similar (Whitehead and Peakall, 2009; Wetzel and Whitehead, 2020). Whether plants divert more chemodiversity into their FVOCs or stored toxins might depend on the individual needs of the plant within its ecological niche and the plethora of visitors with which it must interact. For example, *Prunus dulcis* (almond) shows a high diversity in pollen toxins but a low FVOC diversity (Supplementary data Table S3). Almond plants depend on generalized bees for pollination early in the season, when frost damage poses a danger (Henselek *et al.*, 2018). Given that almond flowers are visited by many bee pollinator species, the plants might use more defence chemicals that enhance their resistance towards florivory, but fewer FVOCs that are adequate to attract generalized bee pollinators to ensure rapid and effective pollination of the flowers (Henselek *et al.*, 2018).

In our second meta-analysis, the pollen protein concentration tended to be higher when the number of pollen toxins was higher (marginally not significant; Spearman correlation, ρ = 0.468, *P* = 0.06, *n* = 16; Fig. 1G). However, no significant relationship was found between the protein concentration and the number of toxin classes in pollen (Spearman correlation, ρ = 0.26, *P* = 0.33; Fig. 1H). A high number of potential toxins in pollen might be beneficial to defend nutrient-rich pollen more effectively. Losses of pollen by herbivory would be costly for the plants. These findings thus support the optimal defence hypothesis (McKey 1974; Rhoades and Bergdahl 1981; McCall and Irwin, 2006; McCall and Fordyce, 2010) in pollen, i.e. nutrient-rich, valuable pollen is better defended than nutrient-poor pollen. Testing other hypotheses for plant chemical defences in flowers would require the generation of additional and more comprehensive data from flowers and visitor behaviour (McCall and Irwin, 2006).

Chemodiversity of FVOCs could, potentially, also have developed as a signal to flower visitors about the reward quality of flowers (Deisig *et al.*, 2006; Knauer and Schiestl, 2015; Burdon *et al.*, 2020). If FVOC chemodiversity is related metabolically to floral reward composition, it might represent an honest signal to visitors about reward quality, including nutrition and toxicity. Alternatively, plants might have used FVOC chemodiversity as a meta-signal to generalist pollinators, independent of the plant species. More research is needed on the impact of chemodiversity on generalist and specialist flower visitors. Among different floral parts, more data could be found for pollen quality in terms of nutrient and defence compounds, and it might also be interesting to study such traits in nectar, sepals and petals. Across all studies reporting on compound richness, the number of FVOCs and toxins found in each study depends on instrument sensitivity and sampling techniques used in the respective study and might thus be biased. With longer sampling times, better sampling quality and more sensitive equipment, more compounds can be detected, increasing the observed richness. This limitation needs to be considered when interpreting the data.

More comprehensive approaches, e.g. regarding the multimodal floral ‘Gestalt’ including FVOC, CO_2_, gustatory and visual cues, as done recently (Raguso and Willis, 2005; Kaczorowski *et al.*, 2012; Katzenberger *et al.*, 2013; Peach *et al.*, 2019), are needed. According to the efficacy back-up hypothesis, the availability of multimodal cues is costly and might deliver redundant information if all cues are salient. However, like the repetition of information in another language, multimodal cues can enhance decision-making (Kulahci *et al.*, 2008) and are particularly decisive for host finding in situations when one cue blurs owing to environmental conditions, such as wind (Lawson *et al.*, 2017; Telles *et al.*, 2017). Correlations between olfactory and contact cues can also be expected for other plant organs apart from flowers within plant individuals. Chemodiversity can be calculated at different levels and for different organs or tissues. Further investigation is needed on the potential use of chemodiversity as a multimodal cue, acting via olfaction, gustation and, occasionally, also visual cues (Kulahci *et al.*, 2008), in addition to the information that it might provide about host plant quality for different types of interaction partners.

## CONCLUSION

The dynamics and plasticity of floral chemistry and insect chemosensation are expected to play a major role in the evolution of plant–insect relationships (Schiestl, 2015). Our review and meta-analyses summarized the essential findings on the role and complexity of various floral chemical displays, including FVOCs, pollen nutrients and pollen toxins, and the impacts that these have on flower visitors, such as pollinators and florivores. Although the number of studies that consider both pollinators and florivores is limited, we could highlight that some FVOCs, such as linalool and methyl salicylate, are rather attractive for pollinators but repellent for florivores. Nevertheless, there are more shared FVOCs that are attractive than repellent for both pollinators and floral herbivores, increasing the dilemma for plants regarding whom to attract. Chemodiversity is increasingly considered as an important trait mediating interactions within and among species (Slinn *et al.*, 2018; Wetzel and Whitehead, 2020; Müller and Junker, 2022; Ziaja and Müller, 2023) and has been found to play an important role in mediating interactions with flower visitors (Egan *et al.*, 2018; Eilers *et al.*, 2021). Future research should investigate the chemodiversity of different tissues within flowers to gain a better understanding of metabolic, ecological and evolutionary forces driving chemical flower displays.

## SUPPLEMENTARY DATA

Supplementary data are available at *Annals of Botany* online and consist of the following. Data S1: A description of the methods used in the review and meta-analyses. Tables S1–S5: The datasets used for the meta-analyses.

mcad064_suppl_Supplementary_Data

mcad064_suppl_Supplementary_Material
